# P-1243. Evaluation of Single and Multiple Dose Safety and Pharmacokinetics of Ledaborbactam Etzadroxil and Ceftibuten-Ledaborbactam Etzadroxil in Healthy Volunteers

**DOI:** 10.1093/ofid/ofae631.1425

**Published:** 2025-01-29

**Authors:** Carlos Fernando de Oliveira, Mary Beth Dorr, Kathryn Lowe, Gregory A Winchell, Paul McGovern

**Affiliations:** Venatorx Pharmaceuticals, Inc, Malvern, Pennsylvania; Venatorx, Malvern, Pennsylvania; Venatorx Pharmaceuticals, Malvern, Pennsylvania; Winchell Pharma Consulting LLC, Norristown, Pennsylvania; Venatorx Pharmaceuticals, Malvern, Pennsylvania

## Abstract

**Background:**

Ledaborbactam etzadroxil (LED-E), a novel oral prodrug that converts to the active β-lactamase inhibitor ledaborbactam (LED), is being developed in combination with ceftibuten (CTB) to address the urgent need for new oral (PO) treatments against drug-resistant gram-negative infections.

**Figure:**
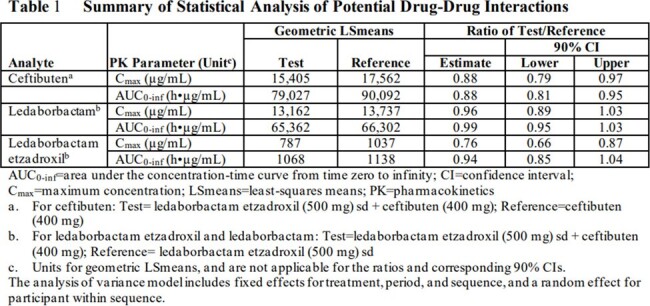

**Methods:**

In part 1, healthy participants (PT) received single (100mg to 1000mg) or multiple (75mg to 500mg q8h for 10 days) PO doses of LED-E or matching placebo (PBO); PTs in Part 2 received single doses of LED-E 500mg, CBT 400mg, and LED-E+CTB 500mg+400mg; Part 3 PT received LED-E+CTB 300mg+400mg, LED-E+CTB 500mg+400mg or PBO q8h for 10 days. Serial blood and urine samples were obtained to determine single dose and steady-state plasma PK of CTB, LED-E, and LED. Safety was assessed by recording adverse events (AE), and changes in laboratory, vital signs, and ECGs.

**Figure:**
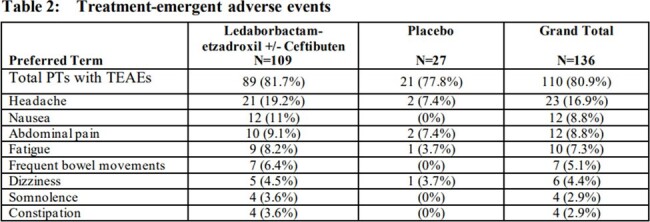

**Results:**

AUC of LED-E was ≤ 2% of exposures of LED across the dose range tested, suggesting extensive conversion of the prodrug to the active drug. LED exposures increased in a generally dose proportional manner across single LED-E doses from 100mg to 1000mg and multiple doses from 75mg to 500mg q8h. The terminal half-life of LED in plasma is approximately 11-12 h, with 30-35% accumulation of LED following q8h dosing of LED-E. Excretion in urine of LED-E derived material was 84.4% over the 8-hour dosing interval at steady state. No clinically relevant changes in the pharmacokinetics of CTB, LED, or LED-E occurred when CTB and LED-E were co-administered compared to each administered alone (Table 1).

The number of participants with treatment-emergent AEs (TEAE) was similar in the LED-E (+/- CTB) (81.7%) and PBO (77.8%) groups. Headache and nausea were the most frequently reported TEAEs in LED-E dosed PTs (Table 2). Most events were mild and only 1 TEAE led to drug discontinuation. No serious adverse events or deaths occurred. No clinical relevant changes were noted in clinical labs, ECGs, or vital signs

**Conclusion:**

LED exposure increased in a dose proportional manner. No clinically relevant drug-drug interaction was observed between LED and CTB When LED-E and CTB are co-administered. LED-E was safe and well tolerated when given with CTB at multiple doses up to 1500mg daily for 10 days.

**Disclosures:**

**Carlos Fernando de Oliveira, MD, PhD, MS**, Venatorx Pharmaceuticals: Stocks/Bonds (Private Company) **Mary Beth Dorr, PhD**, Merck: Stocks/Bonds (Public Company)|Pfizer: Stocks/Bonds (Public Company)|Venatorx: Stocks/Bonds (Private Company) **Kathryn Lowe, MS**, Venatorx Pharmaceuticals: Stocks/Bonds (Private Company) **Gregory A. Winchell, PhD**, Certara: Advisor/Consultant|Merck: Advisor/Consultant|Venatorx: Advisor/Consultant **Paul McGovern, MD**, Venatorx Pharmaceuticals, Inc.: employee|Venatorx Pharmaceuticals, Inc.: Stocks/Bonds (Private Company)

